# Experimental Study of Wireless Monitoring of Human Respiratory Movements Using UWB Impulse Radar Systems

**DOI:** 10.3390/s18093065

**Published:** 2018-09-12

**Authors:** Xiaolin Liang, Yuankai Wang, Shiyou Wu, Thomas Aaron Gulliver

**Affiliations:** 1 Science and Technology on Electronic Test & Measurement Laboratory, The 41st Research Institute of CETC, Xiang Jiang Road 98th, Huangdao District, Qingdao 266555, China; 2 China Electronics Technology Instruments Co. Ltd., Xiang Jiang Road 98th, Huangdao District, Qingdao 266555, China; wykkerry@163.com; 3 The Key Laboratory of Electromagnetic Radiation and Sensing Technology, Institute of Electronics, Chinese Academy of Science, North 4th Ring West Road 19th, Haidian District, Beijing 100190, China; ahwushiyou@126.com; 4 Department of Electrical Computer Engineering, University of Victoria, PO Box 1700, STN CSC, Victoria, BC V8W 2Y2, Canada; agullive@ece.uvic.ca

**Keywords:** impulse ultra-wide band (UWB) radar, automatic gain control (AGC), interested region, human respiratory signals, interested region

## Abstract

This paper analyzes and discusses the capability of human being detection using impulse ultra-wideband (UWB) radar with an improved detection algorithm. The multiple automatic gain control (AGC) technique is employed to enhance the amplitudes of human respiratory signals. Two filters with seven values averaged are used to further improve the signal-to-noise ratio (SNR) of the human respiratory signals. The maximum slope and standard deviation are used for analyzing the characteristics of the received pulses, which can provide two distance estimates for human being detection. Most importantly, based on the two distance estimates, we can accurately judge whether there are human beings in the detection environments or not. The data size can be reduced based on the defined interested region, which can improve the operation efficiency of the radar system for human being detection. The developed algorithm provides excellent performance regarding human being detection, which is validated through comparison with several well-known algorithms.

## 1. Introduction

The ultra-wideband (UWB) radar has been extensively used for the remote sensing of human subjects at short distances through walls and ruins [1]. A human subject can be detected based on his/her chest subtle motion from respiration and heartbeat, which is different from magnetic induction, thermal imaging, X-ray, and some other success-restrictive methods, and cannot be affected by temperature, the material of a non-metal wall, or target clothes [2,3]. UWB radar usually transmits electromagnetic waves with a large bandwidth (usually larger than 500 MHz) i.e., a short pulse period (usually a nanosecond or a picosecond). Most importantly, compared with the continuous wave radar system, UWB radar can acquire the localization of human subjects with higher accuracy. As a result, the UWB radar is considered to be perfect for localization and detection purposes, especially in natural disaster relief [4,5].

Non-invasive monitoring is more suitable for application in some situations where it is challenging to use complex wired connections, such as burn victims or electrocardiogram (ECG) monitoring for infants [6]. Usually, the microwave Doppler radar is considered an alternative to the non-invasive detection of life signs [7]. However, the Doppler radar cannot better penetrate materials and has the problem of null point [8]. Compared with Doppler radar, one of major advantages of UWB signals is its better capability of penetrating materials.

The UWB monitoring of life signs has been widely studied in the literature [9,10,11,12,13,14] as an alternative method to Doppler radar systems [15,16]. The measurements of life signs in different conditions have been widely studied by researchers [17,18,19]. Lazaro et al. analyzed a mathematical model for life sign detection [6]. The authors proved that the collected signals contain human respiration frequency and heartbeat rate. A filter-based harmonics canceller method is developed to extract heartbeat rate from spectrums using Fourier fast transform (FFT). However, the developed algorithm cannot deal with the random body motions for one time measurement. In [20], Van et al. employed the IR-UWB radar for the detection of human motion and posture change. However, this work doesn’t consider the influence of posture and motion detection on life signs. An autocorrelation-based method is developed for life sign detection by Khan et al. in [21]. UWB sensors are used for life sign measurements in through-wall conditions [22]. An improved algorithm is developed to estimate the frequency of human respiration based on the wavelet transform [23]. In [24], Schleicher et al. employed an IR-UWB hardware demonstrator to track objects and measure human breathing frequency. However, the authors cannot provide any method for the measurement of human heartbeat rate. The IR-UWB technology is utilized to alert medical personnel whenever to monitor vital signs and acquire the position of a patient [25]. The system for respiration frequency and heartbeat rate monitoring is discussed, and the results acquired from different sides of human body are obtained and analyzed. However, the authors did not discuss the results from the interval of position change, which may result in invalid measurements [26]. In [27], Yilmaz et al. reviewed various wireless technologies for life sign detection, including breathing frequency, heartbeat rate, blood pressure, and glucose level.

Based on UWB radars, life signs can be extracted accurately even in through-wall conditions, which is most important in natural disaster relief. Demodulation techniques have been used to remove random body movement in quadrature Doppler radar for life sign monitoring [28]. A noise reduction algorithm is presented using an improved ensemble empirical mode decomposition (EEMD) technique, and a separation algorithm based on continuous wavelet transform (CWT) is developed to improve the signal-to-noise ratio (SNR) for detecting respiration frequency and heartbeat rate accurately [29] without considering the strong harmonics of the human breathing signal. An analytical framework for life sign detection is proposed by the authors Venkatesh et al. in [30]. However, no effective algorithm is developed to suppress the harmonics of the human respiration signal when the heartbeat rate and respiration harmonics are located closely. In [31], the magnitude of maxima and minima is used to detect the body motion in the time domain by Javaid, which is inefficient when there is an actual change in the magnitude of the human respiration signal. One elderly-care motion sensor is developed using UWB pulses by Ota et al. [32]. In [33], the body state of the human is monitored using IR-UWB radar by Li et al., but a strategy to overcome the effect of motion on vital sign measurements was missing in these references. Immoreev et al. introduced the practical applications of UWB radar in [34]. The through-wall UWB radar is used to acquire respiration frequency and heartbeat rate, which is operated within a Federal Communications Commission (FCC) mask [35]. The authors Yarovoy et al. use the UWB radar to detect human subjects in a complex environment in [36]. In [37], the spectrums of human respiration are used to detect a human being behind a wall based on UWB radar. However, most algorithms only work well at short distances, and thus cannot be used effectively in complex environments. Therefore, one alternative method has to be required for life detection.

This paper proposes a novel algorithm for human being detection using the UWB impulse radar system. The contributions of the paper are summarized as:

(1) The multiple automatic gain control (AGC) technique is employed to enhance the strength of the respiratory signals of human beings, which can better enhance human respiratory signals and reduce the noise based on the used gain values.
(2) Two filters with seven values averaged are used for improving the SNR of human respiratory signals with one filter performed on the distance direction, and another filter performed on the frequency direction.
(3) Two statistics, including the maximum slope and standard deviation, are used for analysing the characteristics of human respiratory signals. Based on the acquired results, the distance between the radar receiver and human beings can be calculated.
(4) Based on the distance estimate, the interested region containing human respiratory signals can be determined, which can be used to improve the SNR and the accuracy of the frequency estimate of human respiratory movement.
(5) The developed algorithm gives an excellent performance regarding human being detection, which is validated compared with several well-known algorithms.

The four sections are discussed as follows. In Section 2, the IR-UWB radar system is briefly described. Section 3 states the method for human being detection in different environments. Then, the experiment results are presented and analyzed in Section 4. Finally, the conclusion and some proposals for future work are provided in Section 5.

## 2. Experimental Statement

**Figure 1:**
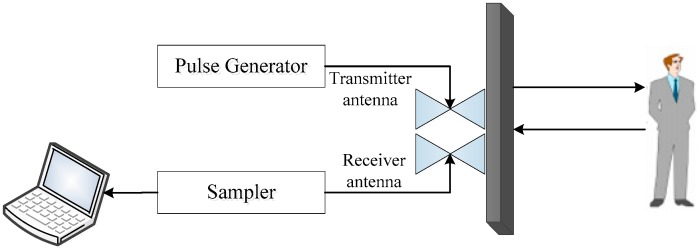
The experimental setups for human being detection.

Different measurements take place in the China National Fire Equipment Quality Supervision Center, and the Institute of Electronics at the Chinese Academy of Sciences. The experimental setups for human being detection are shown in Figure 1. The employed UWB radar for data acquisition is installed on a desk, which contains one receiver antenna and one transmitter antenna. The key parameters for the UWB radar are given in Table 1. Up to 512 pulses can be collected within 17.6 s based on the used UWB radar.

**Figure 2:**
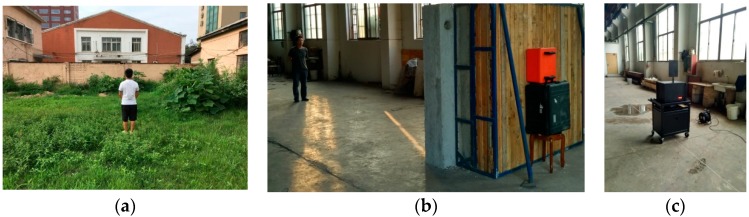
The conducted experiments in through-wall conditions (**a**) outdoors; (**b**) indoors; and (**c**) on the actuator.

To show the fundamental problem of human being detection, two male volunteers served as human beings, as shown in Figure 2, and the experiments in the research were conducted in different conditions.

**Figure 3:**
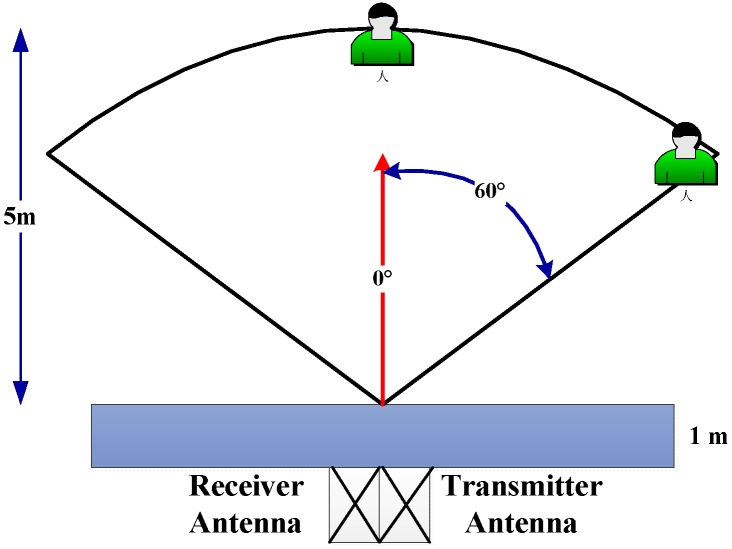
The conducted azimuth experiments.

The first experiment was conducted as shown in Figure 2; one male volunteer served as the detection object. The volunteer faced the radar directly with 0° azimuth between the volunteer and the UWB radar, as shown in Figure 3. The distance from the radar to the volunteer was 3 m, 6 m, and 9 m, respectively.

As shown in Figure 2b, when the second experiment was conducted, another male volunteer served as the detection object. The volunteer faced the radar straight with 0° azimuth between the volunteer and the UWB radar, as shown in Figure 3. The distance from the radar to volunteer was 4 m, 7 m, 10 m, and 12 m, respectively.

As shown in Figure 2c, the third experiment was also conducted. One actuator was used as the detection object, which can rotate at a uniform speed with 3-mm amplitude and a 0.3333-Hz frequency. The actuator faced the radar straight, with 0° azimuth between the actuator and the UWB radar as shown in Figure 3. The distance from the radar to actuator was 4 m, 7 m, 10 m, and 12 m, respectively.

As shown in Figure 3, we conducted the fourth experiment. The volunteer faced the radar straight, with 60° azimuth between the volunteer and the UWB radar. The distance from the radar to the volunteer was 6 m.

**Figure 4:**
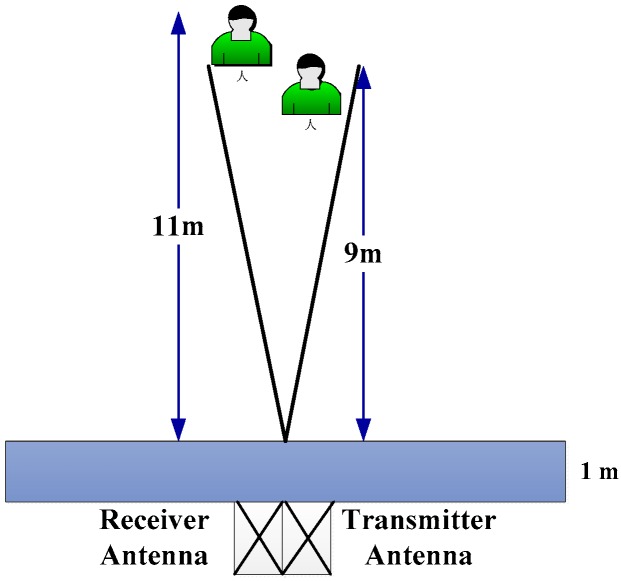
The conducted two-target detection experiments.

Figure 4 shows the conducted experiments in which two volunteers served as the detection subjects. One volunteer is at a distance 10 m away from the radar receiver; another volunteer is 12 m away from the radar receiver. For all of these conducted experiments, the wall between the UWB radar and the detection objects is one meter in thickness. 

## 3. Developed Algorithm

**Figure 5:**
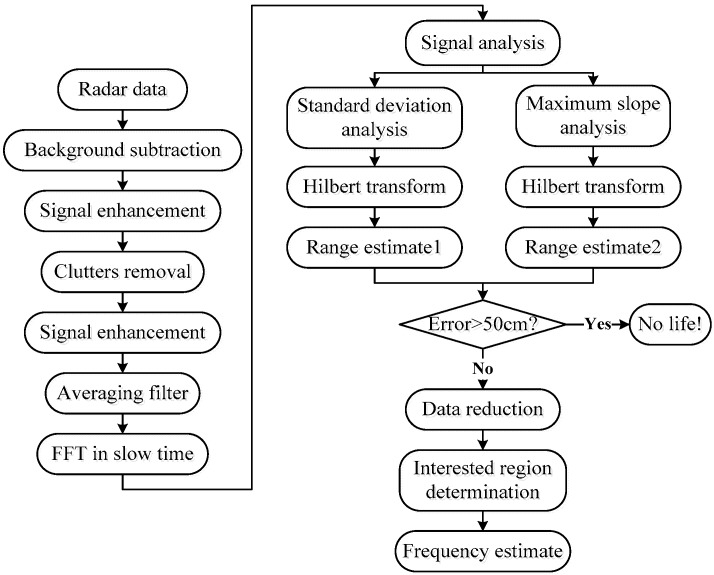
Flowchart of the developed human being detection method.

This section discusses the developed algorithm for human being detection using the UWB impulse radar. The details of the developed algorithm are shown in Figure 5.

### 3.1. Clutters Suppression

To estimate this static clutter, the time-invariant background signal has to be estimated and subtracted from the measured radar data **A**. The received radar data are collected in digital form, which is an *M*×*N* matrix. Several approaches have been proposed for estimating this static clutter. The exponential averaging algorithm is employed for removing the static clutter based on the time-variant weighted coefficient λ, which can be adjusted between 0 and 1. Based on the exponential averaging algorithm, we can acquire the following results [38]:

(1) $$B_{n}\left(m\right)=\lambda_{n}\left(m\right)\times A_{n-1}\left(m\right)+\lambda_{n}\left(m\right)\times A_{n}\left(m\right)$$

According to [39], λ = 0.95 has a reasonable balance between background attenuation and micromovement retention.

Meanwhile, one bandpass filter is performed on Equation (1) with the normalized cutoff frequency 0.1037 for the low pass filter and 0.0222 for the high pass filter, which all are from the Butterworth filters. Using the band pass filter, we can obtain the resultant matrix **C**.

### 3.2. Signal Enhancement

As an adaptive system, the automatic gain control (AGC) technique has been extensively applied in many electronic devices. Using the fed levels of the averaged output signals, we can adjust the gain values to an appropriate level for a range of input signal levels. One of the typical applications using the AGC algorithm is to improve the strength of weak signals in radar systems. Through adjusting the gain values based on the calculated power of the signals in a given window with the width *w*, the key idea of the AGC technique is to compare the acquired gain values and a chosen maximum *g*_max_, which is formally given by:

(2) $$g_{\mathrm{mask}}=\left\{ \begin{array}{ll} g_{\max} & g_{\mathrm{norm}}\left[i,n\right]\geq g_{\max}\\ g_{\mathrm{norm}}\left[i,n\right] & g_{\mathrm{norm}}\left[i,n\right]<g_{\max} \end{array}\right.$$

where n=0,2,⋯,N−1, i=0,1,⋯,M−w, and $g_{\mathrm{norm}}\left[i,n\right]$ is the normalized gain value, which is:

(3) $$g_{\mathrm{norm}}\left[i,n\right]=\frac{g[i,n]}{g_{\min}[i,n]}$$

where $g_{\min}\left[i,n\right]$ is the minimal gain value acquired from all of the *i* values for each *n*, which is:

(4) $$g\left[i,n\right]=\frac{w}{e[i,n]}$$

where e[i,n] is the signal power in a window with the length *w*, which is:

(5) $$e\left[i,n\right]=\sqrt{\sum_{k=i}^{w+i-1}C{\left[k,n\right]}^{2}}$$

Using the AGC method, *g*_max_ can be predetermined based on the calculated gain values. We can acquire the following results:

(6) $$D\left[i,n\right]=C\left[i,n\right]\times g_{\mathrm{mask}}\left[i,n\right]$$

### 3.3. SNR Improvement

To improve SNR of the life signs data, one effective filter with 12 values averaged is presented, which is performed on the columns of Equation (6). We can acquire the following results [40]:

(7) $$E\left[k,n\right]=\frac{1}{7}\sum_{m=6k}^{7k-1}D\left[m,n\right]$$

where k=1,⋯,⌊M/7⌋. ⌊M/7⌋ is the maximal integer values, which is less than M/7.

### 3.4. Spectrums Analysis

By performing the autocorrelation function on the *mth* slow time signal *x_m_*(*n*) in Equation (7), the influences caused by the zero-mean random noise are too weak for human being detection. Especially for the non-periodic noise, the autocorrelation function is prone to zero. As a result, the autocorrelation function is used to improve the weak respiratory signals. Further, to extract the spectrums of human respiratory signals, FFT is performed on the results from the autocorrelation function in slow time direction. We can acquire the resultant matrix, which is distance frequency matrix **F**.

**Figure 6:**
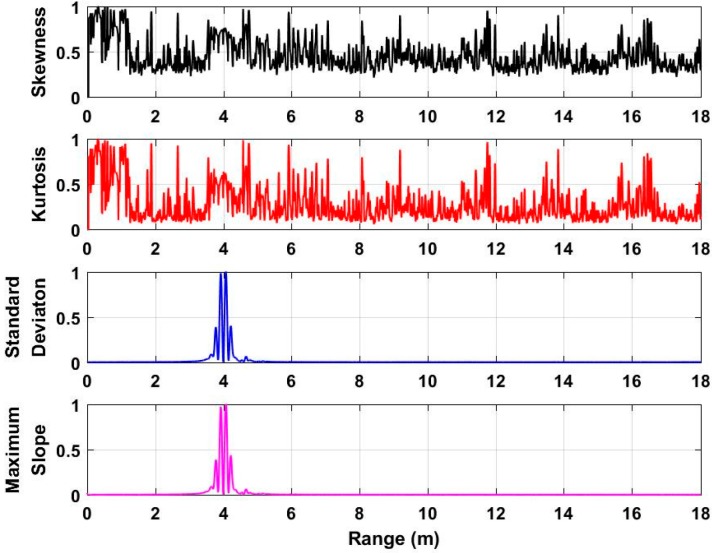
The analyzed characteristics based on the acquired data at a distance of 4 m.

To acquire the distance between human beings and UWB radar, the characteristics including the skewness [41], kurtosis [42], standard deviation [43], and the maximum slope [44] of each slow time signal in **F** are analyzed and discussed. Based on the acquired data from the second experiment at a distance of 4 m, the analyzed characteristics with normalized values are shown in Figure 6. Based on the analyzed results, we can see that the standard deviation and maximum slope can better be used for distance estimation compared with the skewness and kurtosis. As a result, this paper employs the standard deviation and maximum slope as factors to acquire the distance from the radar to the human being. The distance estimate corresponding to the maximal value of the calculated standard deviation can be acquired, which is considered as *τ*_1_. Meanwhile, the distance estimate corresponding to the maximal value of the calculated maximum slope can be acquired, which is considered as *τ*_2_. The acquired two distance estimates can be used as thresholds for human being detection, which will be discussed in the following subsection.

### 3.5. Object Detection

To determine whether there are human beings in the detection environment, the absolute error calculated from the two distance estimates are acquired, which is given by:

(8) $$\delta =\tau_{1}-\tau_{2}$$

**Figure 7:**
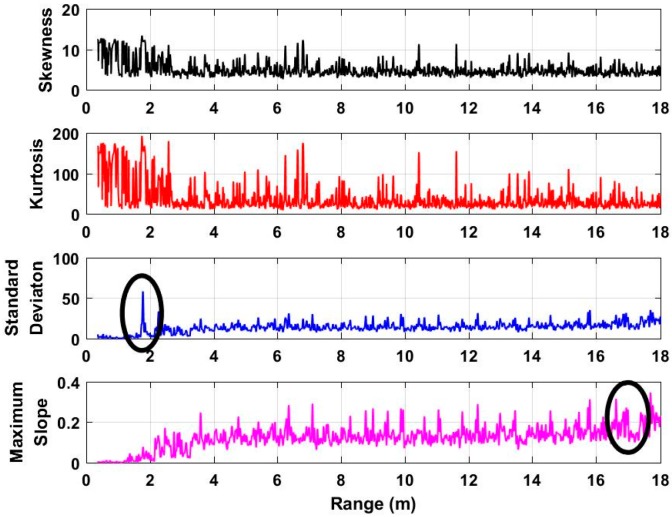
The analyzed characteristics based on the data without human beings in the experiment.

Using Equation (8), we can determine whether there are human beings in the detection environment or not through considering the threshold as a fitted value, such as 50 cm. Based on the fitted threshold, there are no human beings in the detection environment when *δ* > 50 cm. However, there are human beings in the detection environment when *δ* ≤ 50 cm. Based on the acquired data when there are no human beings in the environment, the analyzed characteristics are shown in Figure 7. We can see that the error from the two estimated distances is much more than several meters. Further, based on the acquired 72 data sets, the calculated errors are all in the level of meters. As a result, 50 cm can be considered as a perfect threshold for human being detection.

### 3.6. Interested Region Determination

**Figure 8:**
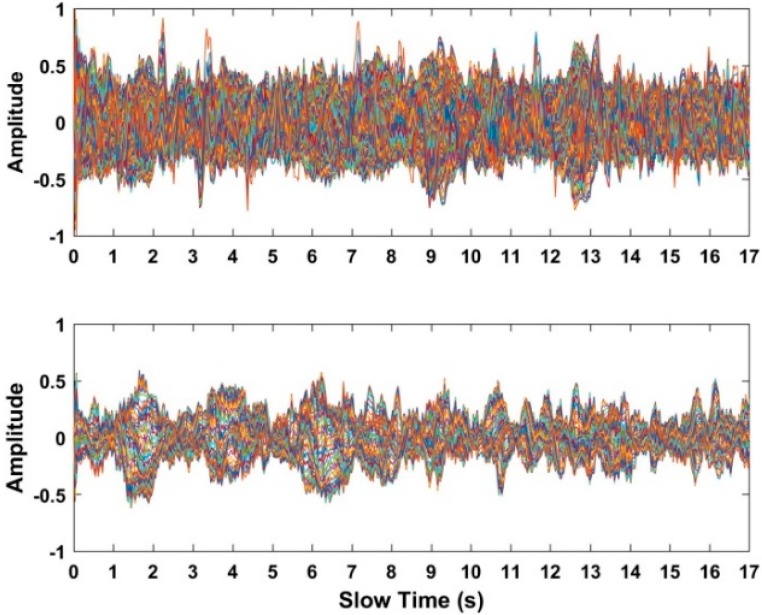
The acquired data from one human being at a distance of 12 m.

To estimate the frequency of human respiratory signals, the interested region containing the signals of human respiration is determined in this subsection based on the distance estimate *τ*_1_. In this subsection, the interested region is defined as the scope [*υ* − 20, *υ* + 20], where *υ* corresponds to the index of the distance estimate *τ*_1_ in the distance frequency matrix. The acquired data and the signals in the interested region are shown in Figure 8 based on the data at a distance of 12 m. As shown in Figure 8, human respiratory signals are covered with noise with larger strength, which makes it challenging to acquire the frequency when all of the data are considered. However, we can see the radar pulses modulated by human respiratory movement when only the signals in the interested region are considered. As a result, the signals in the defined interested region can better improve the SNR, which can make the frequency easier to estimate. Most importantly, the interested region can reduce the data size, which can improve the operation efficiency of the radar system.

### 3.7. Frequency Estimate

Since the frequency of human respiratory signals is confined to a narrow frequency distance, one selected frequency window within 0.1–0.8 Hz is used for removing clutters and harmonics in very high and very low frequencies. Based on the employed frequency window, for each frequency signal *y_m_*(*n*) in **F**, we can acquire the following results:

(9) $$g_{m}=y_{m}\left(n\right)⊙\Psi ,m\in \left[\upsilon -20,\upsilon +20\right]$$

where Ψ denotes the added frequency window.

To acquire the frequency of human respiratory signals, the accumulated spectrums are considered, which are given by:

(10) $$\rho =\sum_{m=\upsilon -20}^{\upsilon +20}g_{m}$$

Usually, the maximal value in Equation (10) is defined as the frequency of human respiratory signals.

## 4. Results and Discussion

This section discusses the developed algorithm for human being detection. 

### 4.1. Clutters Suppression

**Figure 9:**
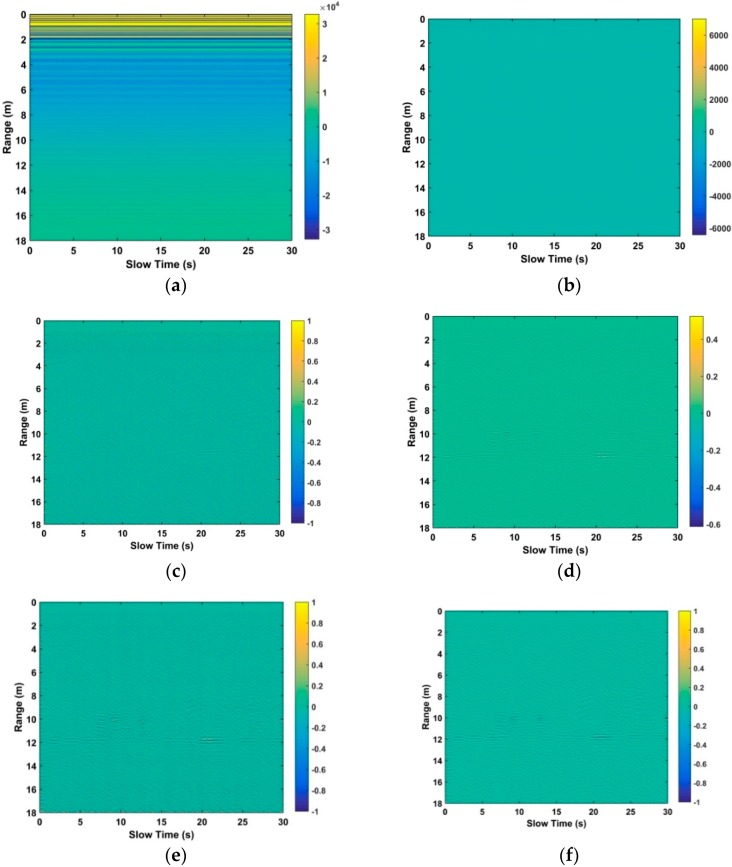

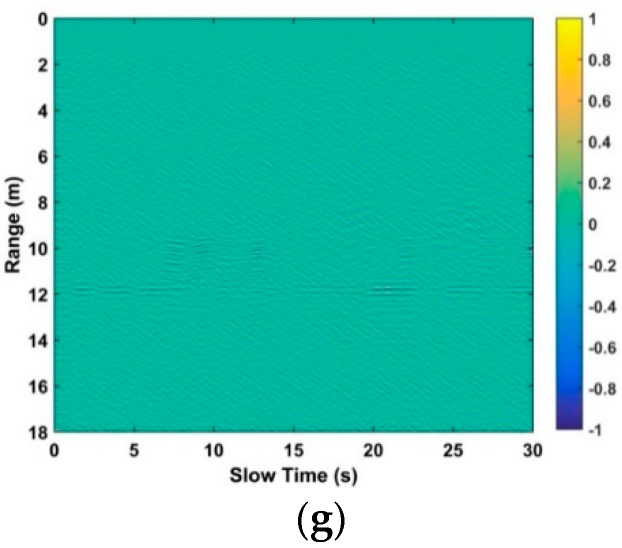
The resultant matrix from the (**a**) radar receiver; (**b**) static background suppression; (**c**) automatic gain control (AGC); (**d**) band pass filter; (**e**) AGC; (**f**) averaging filter in distance direction; and (**g**) averaging filter in slow time direction.

This subsection analyzes the capability of clutters suppression based on the received data from human beings at a distance of 12 m away from the UWB impulse radar. Figure 9a shows the acquired distance–time (slow time) matrix from the radar receiver, which indicates that human respiratory signals are covered with different noises with larger amplitude. When using the radar data, human respiratory signals are challenging to identify due to the existing noises. The resultant matrix obtained from the used static background suppression algorithm is shown in Figure 9b.

Figure 9c shows the resultant matrix obtained from the used AGC algorithm, which is performed for the first time. We can see that human respiratory signals are improved and the clutters and noises are preliminarily suppressed. As shown in Figure 9d, the resultant matrix is acquired using the employed band pass filter, which indicates that human respiratory signals are further improved. Figure 9e shows the resultant matrix obtained from the used AGC algorithm, which is performed for the second time. The resultant matrix is shown in Figure 9f using the averaging filter performed in the slow time direction. Figure 9g shows the resultant matrix based on the averaging filter performed in the distance direction. Compared with Figure 9a, we can see that the human respiratory signals are improved effectively, which make human beings easier to identify.

### 4.2. Distance Estimate

**Figure 10:**
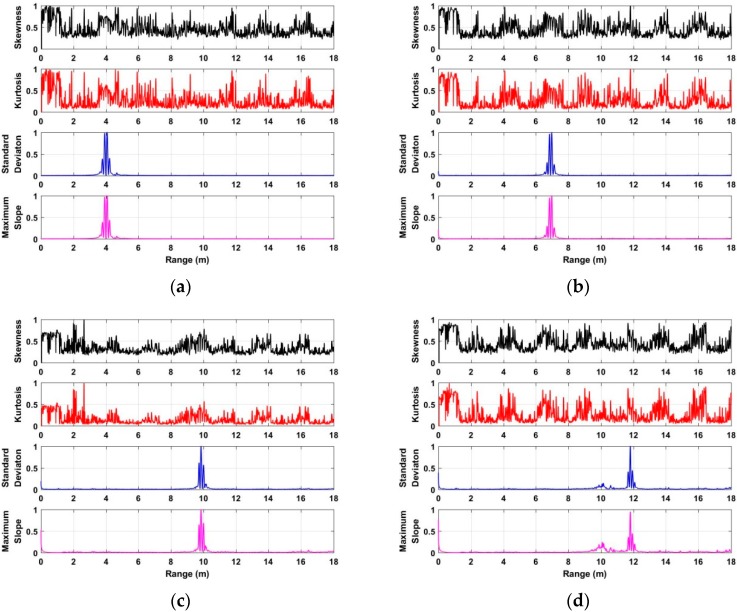
The distance estimates based on the acquired data from one human being at a distance of (**a**) 4 m; (**b**) 7 m; (**c**) 10 m; and (**d**) 12 m indoors.

**Figure 11:**
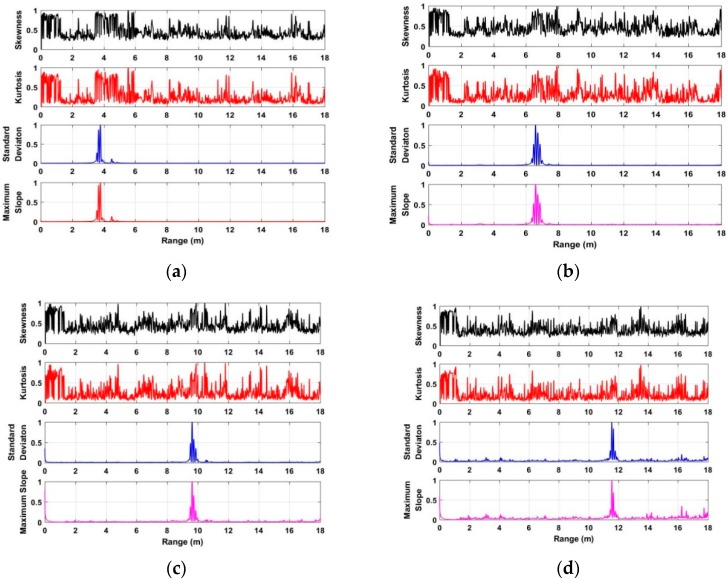
The distance estimates based on the acquired data from the actuator at distances of (**a**) 4 m; (**b**) 7 m; (**c**) 10 m; and (**d**) 12 m indoors.

**Figure 12:**
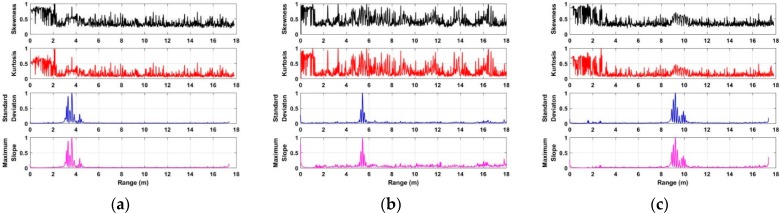
The distance estimates based on the acquired data from a human being at distances of (**a**) 3 m; (**b**) 6 m; and (**c**) 9 m outdoors.

The performances of distance estimate based on the developed algorithm are analyzed and discussed using the data acquired in different environments. Based on the radar data acquired from one human being at distances of 4 m, 7 m, 10 m, and 12 m indoors, the distance estimates are shown in Figure 10. Using the developed algorithm, Figure 11 shows the distance estimates based on the radar data acquired from the used actuator at distances of 4 m, 7 m, 10 m, and 12 m. The distance estimates using the data acquired from one human being at distances of 3 m, 6 m, and 9 m are shown in Figure 12. All of the distance estimates mentioned above were acquired from the data with 0° azimuth between the human being and the UWB radar.

**Figure 13:**
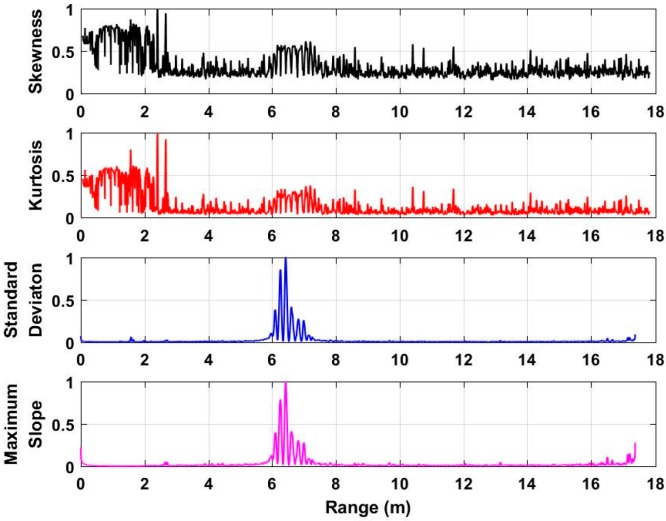
The distance estimates based on the acquired data from one human being at a distance of 6 m indoors with 60° azimuth.

Figure 13 shows the distance estimate acquired from the data at a distance of 6 m away from the radar, and the azimuth is 60° between the human being and UWB impulse radar indoors. Based on the results of distance estimates as shown in Figure 10, Figure 11, Figure 12 and Figure 13 we can see that the distance between the human being and UWB radar can be calculated accurately via analyzing the characteristics of radar data in the conducted experiments, especially in long-distance conditions. Meanwhile, we can see that all of the errors that were calculated from two distance estimates via analyzing the maximum slope and standard deviation of radar data for each given distance are less than 50 cm.

In this subsection, three algorithms are employed as references, including the constant false alarm ratio (CFAR) algorithm [45], advanced method (AM) [46], and the multiple higher order cumulant (MHOC) method [47] to validate the performances of the developed method.

Table 2 shows the errors of the distance estimates based on the four algorithms. We can see that the developed method can provide the highest accuracy compared with the other three methods. All of these results indicate the better performance of the developed algorithm on distance estimates.

### 4.3. Frequency Estimate

The performances of the frequency estimate of human respiratory signals based on the developed algorithm are analyzed and discussed using the data acquired in different environments.

**Figure 14:**
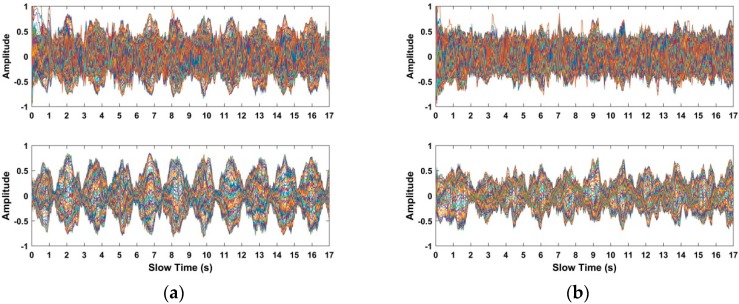

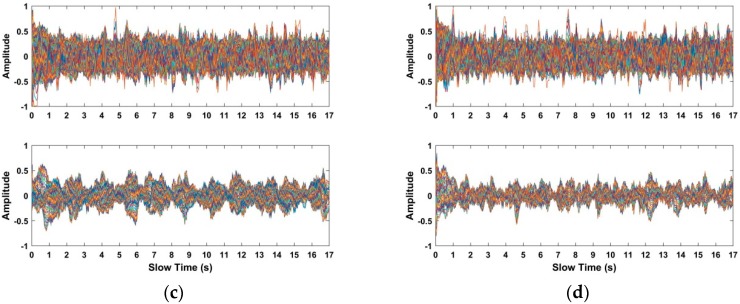
The selected signals in the interested region based on the acquired data from the used actuator at distances (**a**) 4 m; (**b**) 7 m; (**c**) 10 m; and (**d**) 12 m indoors.

**Figure 15:**
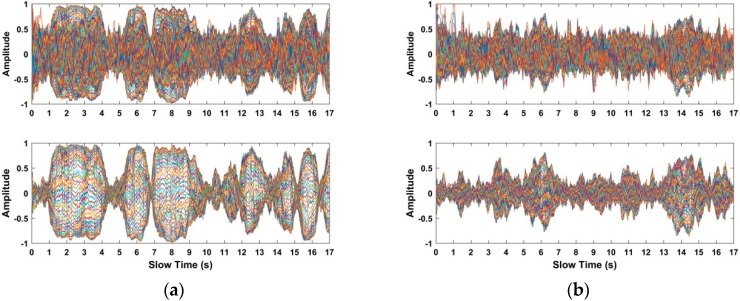

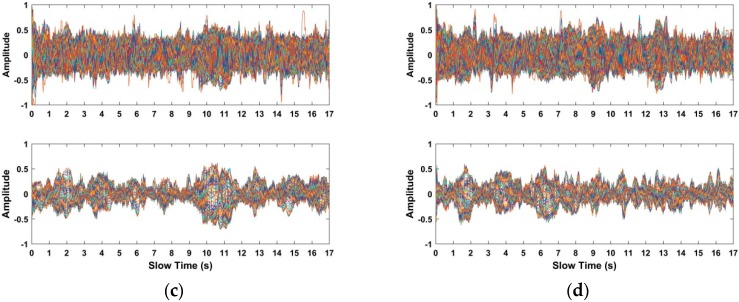
The selected signals in the interested region based on the acquired data from the actuator at distances of (**a**) 4 m; (**b**) 7 m; (**c**) 10 m; and (**d**) 12 m indoors.

Based on the data acquired from the used actuator at distances of 4 m, 7 m, 10 m, and 12 m indoors, the received data and the selected signals in the interested region are shown in Figure 14. Meanwhile, using the radar data acquired from a human being at distances of 4 m, 7 m, 10 m, and 12 m indoors, the received radar data and the selected signals in the interested region are shown in Figure 15. The up figures show the signals from the received radar data, while the signals in the interested region are shown in the following figures. Compared with the up figures, we can see that the SNR of human respiratory signals is improved and the noise affecting human being detection is suppressed effectively, which can make the human being easier to identify.

**Figure 16:**
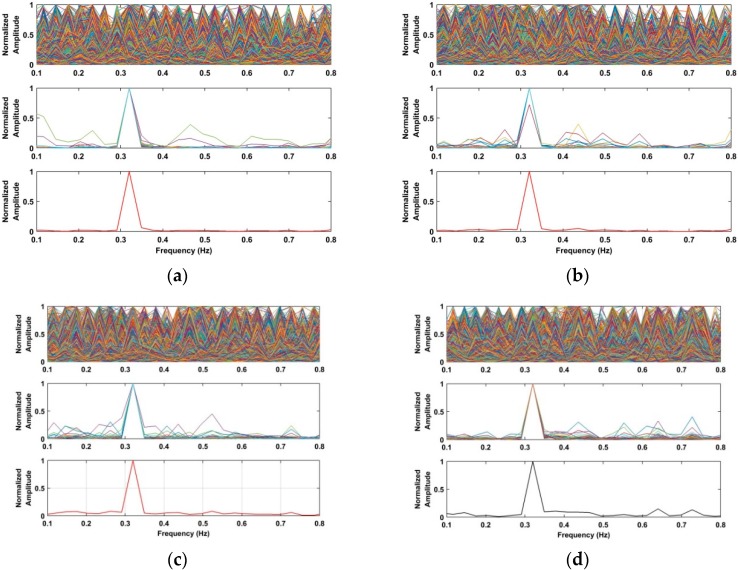
Frequency estimates of the used actuator based on the acquired data at distances of (**a**) 4 m; (**b**) 7 m; (**c**) 10 m; and (**d**) 12 m indoors.

To validate the accuracy of the frequency estimate, the data acquired from the used actuator at different distances, including 4 m, 7 m, 10 m, and 12 m indoors, are employed as references. The frequency estimates of the used actuator at different distances based on the selected signals in the interested region are shown in Figure 16. Based on the acquired results, the frequency estimates of the actuator can be calculated accurately. Meanwhile, the corresponding frequency estimates acquired from the four different algorithms are shown in Table 3, which indicate that the proposed algorithm provides the highest accuracy of the frequency estimates compared with the other three methods.

### 4.4. Detection of Two Human Beings

**Figure 17:**
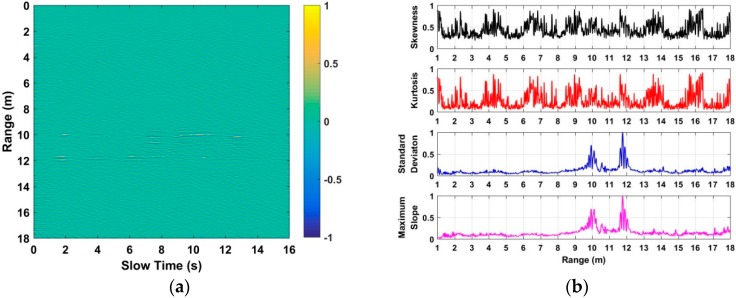
Results of the detection of two human beings (**a**) after removing clutter; and (**b**) distance estimates.

This subsection validates the capability of two-target detection based on the acquired data. Figure 17 shows the detection results based on the developed method using the data from two human beings in the environment. One human being is at a distance of 10 m, while another is at a distance of 12 m away from the UWB impulse radar. The resultant matrix after removing the clutter is shown in Figure 17a,b shows the distance estimates of the two human beings corresponding to the first two peaks in the calculated standard deviation and maximum slope values.

**Figure 18:**
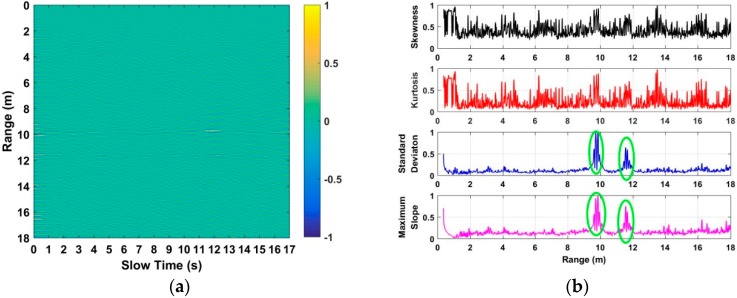
Results of the detection of two targets (**a**) after removing clutter; and (**b**) distance estimates.

Further, Figure 18 shows the detection results based on the developed method using the data from two actuators in the environment. One actuator is at a distance of 10 m, while another is at a distance 12 m away from the UWB impulse radar. The resultant matrix after removing the clutter is shown in Figure 18a,b shows the distance estimates of the two actuators corresponding to the first two peaks in the calculated standard deviation and maximum slope values. From the acquired results, the excellent performance of the proposed algorithm is validated evenly for the two targets.

## 5. Conclusions

This paper proposes a novel algorithm for human being detection using the ultra-wideband (UWB) impulse radar systems. The multiple automatic gain control (AGC) technique is employed to enhance the strength of the respiratory signals of human beings. Two filters with seven values averaged are used for improving the signal-to-noise ratio (SNR) of human respiratory signals. The maximum slope and standard deviation are used for analyzing the characteristics of human respiratory signals. Based on the acquired results, the distance between the radar receiver and human beings can be calculated. Based on the distance estimate, the interested region containing human beings can be determined, which can be used to improve the SNR and the accuracy of the frequency estimate of human respiratory movement. Our future work will mainly focus on how to extract human respiratory signals when they are in motion such as walking and running. Further, we will continue to analyze how to identify human beings from other living animals.

## Figures and Tables

**Table 1:** Parameters for the ultra-wideband (UWB) impulse radar.

Parameter	Value
center frequency	400 MHz
bandwidth of the pulse	400 MHz
transmitted signal amplitude	50 V
pulse repeat frequency	600 KHz
number of averaged values	30
time window	124 ns
number of samples	4092
input bandwidth of the analog to digital converter (ADC)	2.3 GHz
ADC sampling rate	500 MHz
ADC sample size	12 bits
receiver dynamic range	72 dB

**Table 2:** Distance estimation with four different methods. CFAR: constant false alarm ratio, MHOC: multiple higher order cumulant, AM: advanced method.

Methods		4 m	7 m	10 m	12 m
CFAR	Error (m)	0.36	3.47	7.32	8.78
Proposed	Error (m)	0.15	0.17	0.21	0.24
MHOC	Error (m)	0.65	2.93	2.56	8.38
AM	Error (m)	0.34	3.46	5.62	4.25

**Table 3:** Frequency estimates with the two methods. FFT: Fourier fast transform.

Method	4 m		7 m		10 m		12 m	
	Hz	Deviation (%)	Hz	Deviation (%)	Hz	Deviation (%)	Hz	Deviation (%)
FFT	0.116	65.2	0.126	62.00	0.137	58.66	0.112	66.22
Proposed	0.32	3.99	0.32	3.99	0.32	3.99	0.32	3.99
MHOC	0.349	4.74	0.116	65.10	0.116	65.14	0.087	73.89
AM	0.187	43.89	0.087	73.89	0.087	73.89	0.087	73.89
